# Hepatitis C Nucleic Acid Test Positive (NAT+) Solid Organ Consent Rates Are Highest in Patients Listed for Liver Transplant and With an English Language Preference

**DOI:** 10.1111/ctr.70186

**Published:** 2025-06

**Authors:** Sachiko M. Oshima, Alice Parish, Jacqueline B. Henson, Mariya Samoylova, Donna Niedzwiecki, Lisa McElroy, Lindsay King, Julius M. Wilder, Kara Wegermann

**Affiliations:** 1 Division of Gastroenterology, Department of Medicine, Duke University Health System, Durham, North Carolina, USA; 2 Department of Biostatistics & Bioinformatics, Duke University, Durham, North Carolina, USA; 3 Department of Surgery, Duke University Health System, Durham, North Carolina, USA

**Keywords:** disparities, hepatitis C, infection and infectious agents, liver disease, organ allocation, transplant social worker, viral

## Abstract

**Background:**

Transplantation of hepatitis C virus (HCV) nucleic acid (NAT) positive organs is associated with shorter time to transplant and decreased risk of death on the waiting list. Treatment for HCV post-transplant is well-tolerated, successful, and leads to similar transplant outcomes to patients transplanted with HCV NAT− organs. Despite these outcomes, not all patients consent to receive HCV NAT+ organs, and factors associated with consent are not well-known.

**Methods:**

This retrospective single-center study of adult patients listed for heart, liver, lung, and kidney transplant aimed to determine whether sociodemographic and organ-specific disparities exist in consent for HCV NAT+ donor organs.

**Results:**

Of 2788 transplant candidates, 44% (*N* = 1229) consented to receive an HCV NAT+ organ. Patients who designated English as their preferred language were more likely to consent compared to a non-English preference (45% vs. 19%, *p* < 0.001). Consent rates were highest amongst patients listed for liver transplantation compared to kidney, heart, and lung transplants (67%, *N* = 319 vs. 42%, *N* = 602 vs. 38%, *N* = 159 vs. 32%, *N* = 149; *p* < 0.001).

**Conclusions:**

Overall, more efforts are needed to ensure that all patients who may benefit from consenting for HCV NAT+ organs are appropriately educated in their language of choice on the risks and benefits.

## 1 | Introduction

Solid organ transplants are life-saving interventions for individuals with end-organ disease [1], but disparities by sex and race exist along the transplantation continuum of care in the United States [2, 3]. The COVID-19 pandemic further exacerbated this growing crisis with United Network for Organ Sharing (UNOS) reporting a 26.2% national increase in waitlist deaths disproportionately impacting Black patients during the early months of the COVID-19 pandemic [4, 5]. Yet, demand for transplants continues to rise [6], and persistent organ scarcity could further widen these disparities. One potential remedy for organ scarcity is transplantation of Hepatitis C (HCV) nucleic acid (NAT) positive (“HCV NAT+”) organs. In 2015, UNOS mandated reporting of donor HCV NAT status in addition to the previously reported HCV antibody (Ab) testing informing potential recipients of active infection (NAT+) likely to transmit HCV to the recipient versus prior infection (Ab+/NAT−) [7, 8]. The availability of these organs has substantially increased over the past decade due to the tragic rise in opioid overdose deaths [9, 10]. Furthermore, the advent of direct-acting antivirals (DAA) has made post-transplant HCV treatment safe and effective [11].

Although transplantation with HCV NAT+ organs leads to similar outcomes as patients transplanted with HCV NAT negative (NAT−), or uninfected, organs [12–14], barriers to obtaining HCV NAT+ organs exist through the consent process. In the current system, patients must opt-in to accepting HCV Ab+/NAT+ organs prior to transplant. Acceptance of HCV NAT+ organs is associated with faster time to transplant and decreased risk of death on the waiting list [15–19]. Studies have shown that DAA treatment for HCV post-transplant is well-tolerated and successful [12–14, 20, 21]. Therefore, HCV NAT+ organs could be one way alleviate organ shortages in transplantation, but unequal acceptance among groups could widen existing disparities. Factors associated with consent for NAT+ organs and whether this differs for marginalized groups; however, are unknown. The aim of this study was to identify sociodemographic factors associated with consent to receive HCV NAT+ organs. Secondary aims were to identify differences in HCV NAT+ consent across solid organs and to assess if time to transplant differs by HCV NAT+ consent.

## 2 | Methods

### 2.1 | Study Design and Participants

**FIGURE 1 |:**
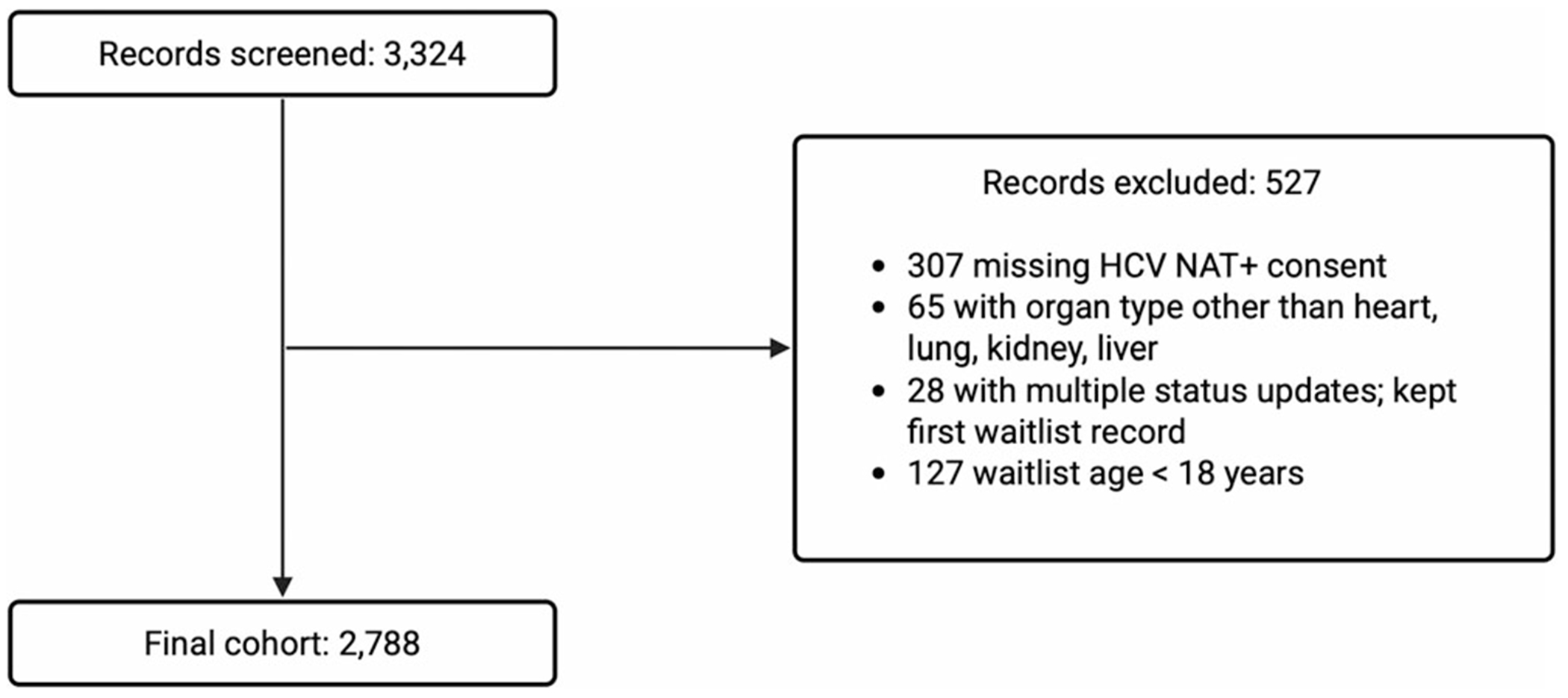
Study inclusion diagram.

This was a retrospective single-center study of adult patients listed for heart, liver, lung, or kidney single organ transplant from January 1, 2018 through June 30, 2022. Patients were excluded if they were missing HCV NAT+ consent in the transplant database (Figure 1). Additional variables extracted from the electronic medical record included age, sex, race-ethnicity, language preference, marital status, insurance status, and baseline HCV Ab/NAT status. Patients’ home address was dichotomized into rural or urban based on county breakdown in the US Office of Management and Budget and US Department of Agriculture Urban Influence Codes with codes 1–2 labeled as urban and codes 3–12 labeled as rural [22, 23]. We were unable to obtain patients’ baseline HCV Ab/NAT status at their time of consent. If patients were listed for solid organ transplant multiple times, consent from their first listing was utilized. The study was approved by the Duke University Institutional Review Board. Data were stored in a report in the electronic health record (Epic Systems Corporation, Verona, WI, USA).

### 2.2 | Patient Consent Processes

Pre-transplant HCV NAT+ consent practices were described by medical directors of the heart, liver, lung, and kidney transplant programs at our center. Consent was obtained by the medical team, as opposed to surgical teams, across transplant fields, and occurred following initial education sessions and consent discussions with transplant coordinators. Patients also had the opportunity to discuss consent with medical providers during the transplant evaluation process. In person Spanish-language interpreters were available, with tablet-based video interpreters for other languages. Consent forms were available in English and Spanish. Each transplant program specified medical criteria that made patients ineligible for HCV NAT+ organs; these patients were not separately identified in our transplant database and therefore were included in the non-consent group in our sample. All other patients were offered HCV NAT+ organs.

### 2.3 | Outcome Measures and Statistical Analysis

The primary outcome was consent to receive HCV NAT+ organs. Descriptive statistics were used to summarize the cohort. Continuous and categorical variables were summarized with median (interquartile range, IQR) and *N* (%), respectively, by HCV NAT+ consent. Differences were calculated using Chi-square or Fisher’s exact tests, as appropriate. A logistic regression model with HCV NAT+ consent as the outcome was performed including race/ethnicity, age, organ type, insurance, marital status, urban/rural, and sex as covariates selected a priori, with odds ratios (OR) and 95% confidence intervals (CI) reported.

To assess if time to transplant differed by HCV NAT+ consent, a series of Fine-Gray competing risk survival models were constructed, accounting for the competing risk of death on the waitlist or delisting. Patients were excluded from this analysis if they were missing time to transplant data. Organ type, race/ethnicity, HCV NAT+ consent, and their interactions, defined a priori, were evaluated. Subgroup models by each organ type were built, also evaluating HCV NAT+ consent, race/ethnicity, and the interaction between the two as covariates in the model. Patients were censored at their last status entry in the electronic health record during the study period if they were still on the waitlist. For all analyses, the threshold for significance was set at a level *α* = 0.05. All statistical analyses were conducted with SAS (SAS Institute, Cary NC, USA).

## 3 | Results

A total of 2788 patients were included in the study, of whom 44% (*n* = 1229) consented to receive an HCV NAT+ organ. The median age of the cohort was 56 years old, and it was 63.1% male. The cohort was 52.3% Non-Hispanic White, 35.5% Non-Hispanic Black, 4.1% Non-Hispanic Other, 2.9% Hispanic or Latino with 5.3% of an unknown race or declined to answer. The majority o the cohort preferred English to other languages (98.5%), had more than a high school education (70.1%), and lived in urban locations (76.8%) compared to rural locations. Most of the patients were insured by Medicare (58.1%), with 34.7% having private insurance 3% having Medicaid, and 4.1% having other insurance. More than half of the cohort was listed for a kidney transplant (51.4%) followed by liver transplant (17.0%), lung transplant (16.7%), and heart transplant (14.9%; Table 1).

### 3.1 | Factors Associated With HCV NAT+ Consent

**FIGURE 2 |:**
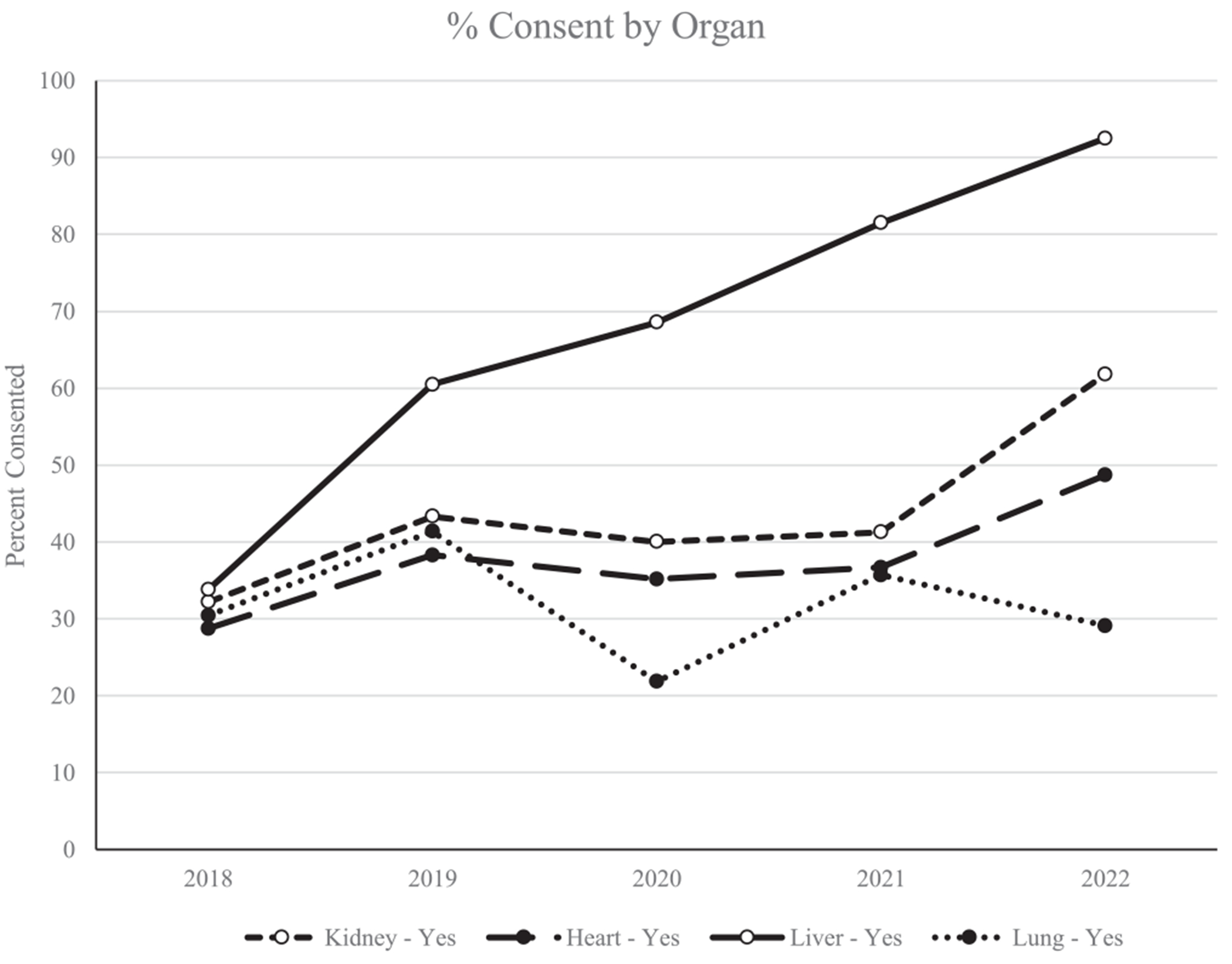
HCV NAT+ consent by organ type.

**FIGURE 3 |:**
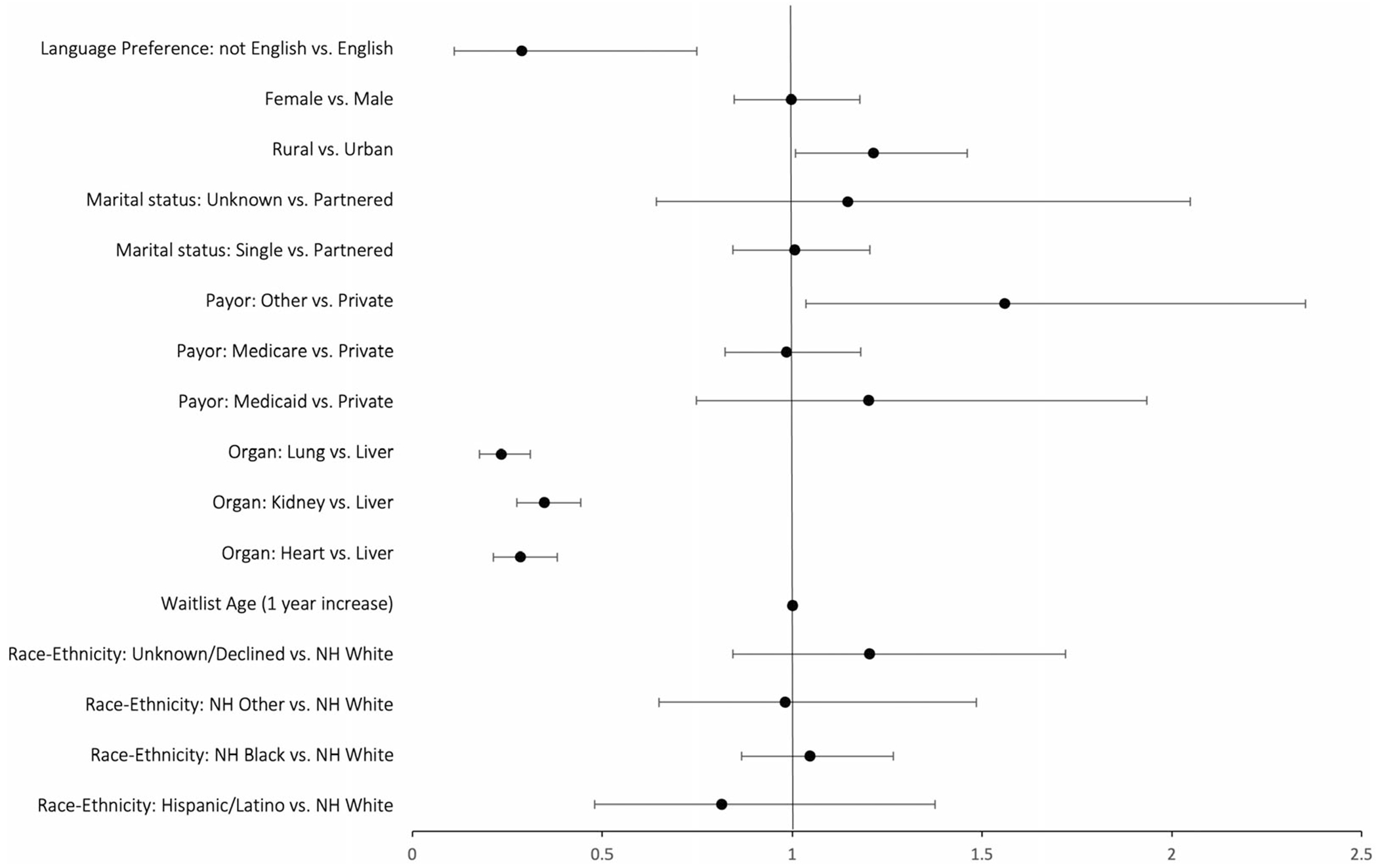
Forest plot of adjusted association of sociodemographic factors with HCV NAT+ consent.

There were no differences in consent for HCV NAT+ organs based on patient age, sex, race-ethnicity, insurance status, education level, or marital status (Table 1). Patients who designated English as their preferred language via their online chart portal were more likely to consent compared to a non-English preference (45% [95% CI: 42.7%, 46.5%] vs. 19% [95% CI: 8.4%, 33.4%]; *p* < 0.001) Consent rates were highest amongst patients listed for a liver transplant (67%) compared to kidney (42%), heart (38%), and lung (32%) transplants (*p* < 0.001; Figure 2). These findings held in a logistic regression model controlling for race/ethnicity age, organ type, insurance, marital status, urban/rural, and sex with patients listed for a heart (OR 0.29, CI 0.21, 0.38), kidney (OR 0.35, CI 0.28, 0.44), and lung (OR 0.24, CI 0.18, 0.31 *p* < 0.0001; Figure 3) transplant less likely to consent for HCV NAT+ organs compared to liver transplant. Consent increased over time, with 32% (*n* = 124) of patients consenting in 2018 compared to 57% (*n* = 200) of patients consenting in 2022 (*p* < 0.001). Patients with home address in rural counties were more likely to consent compared to patients with home address in urban counties (49.1%, *n* = 314 vs. 42.9% *n* = 908; OR 1.23 CI 1.02, 1.48). There was a significant amount of missingness in baseline HCV Ab status (72%, *n* = 2005); of patients who were HCV Ab+ at baseline, 60% (*n* = 24) consented to receive HCV NAT+ organs compared to 47% (*n* = 351) of HCV Ab− patients.

### 3.2 | Time to Transplantation by HCV NAT+ Consent and Organ Type

**FIGURE 4 |:**
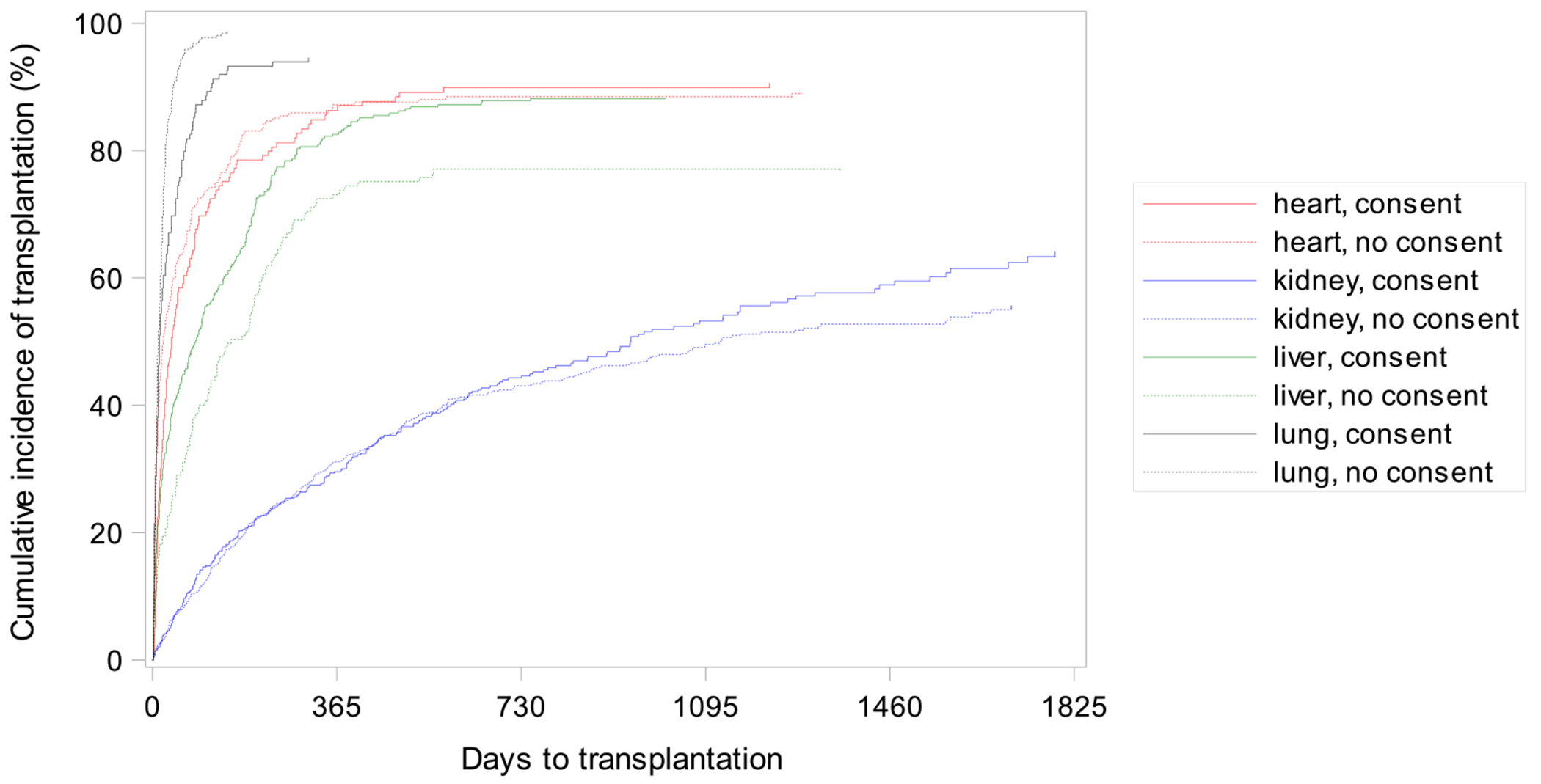
Cumulative incidence function for HCV NAT+ consent by organ type.

There were 2786 patients included in these analyses with two patients excluded due to missing time to transplant data. Of those, 1774 (64%) were transplanted, 535 (19%) remained on the waitlist, and 477 (17%) died or were removed from the waitlist. There was a significant interaction between consent to an HCV NAT+ organ and organ type on time to transplant (*p* < 0.001; Table 2). Patients listed for a liver transplant who did not consent for an HCV NAT+ were less likely to receive a transplant compared to patients who consented (hazard ratio [HR] 0.67, CI 0.54, 0.84). However, for patients listed for a lung transplant, patients who did not consent for an HCV NAT+ organ were more likely to receive a transplant compared to patients who consented to receive HCV NAT+ organs (HR 1.64, CI 1.26, 2.12; Figure 4).

## 4 | Discussion

Solid organ transplantation using donors with HCV has become an increasingly common practice to meet the growing demand for solid organ transplantation [6]. In 2020, the Centers for Disease Control and Prevention recommended that transplant centers assume the responsibility for educating and consenting patients to receive HCV NAT+ organs [24], leading to variation in the consent process between and within transplant centers. Although there are preliminary studies documenting excellent post-transplant outcomes [12, 13, 16, 17] for patients receiving HCV NAT+ organs [14, 20, 21], consent for these organs is not universal, and the characteristics of transplant candidates consenting to receive HCV NAT+ organs are poorly understood [25, 26].

Less than half of the patients in our study (44%) consented to receive HCV NAT+ organs. Notably, the proportion of patients consenting nearly doubled from 2018 to 2022, correlating with increased acceptance of transplanting HCV NAT+ into HCV NAT− patients [8, 17, 27]. Our study is the first to report that HCV NAT+ consent rates were significantly higher in patients who prefer English compared to other languages. Little is known about how linguistic preferences can impact the transplant process, but initial studies in the kidney transplant population found that little educational information is available online in non-English languages [28] and that patients from non-English preferring communities were less likely to complete transplant evaluations and to be waitlisted [29]. This suggests that linguistic barriers may impact patients along the transplant continuum of care. Further studies are needed to explore this potential interaction. With regards to interventions, transplant centers can ensure that educational materials on HCV NAT+ organs are translated into a patient’s preferred language and that translators are consistently available and present during the consent process.

HCV NAT+ consent also differed significantly by organ type with 67% of patients listed for a liver transplant consenting to receive HCV NAT+ organs compared to only 42% of kidney transplant patients, 38% of heart transplant patients, and 32% of lung transplant patients, differences that remained significant after controlling for other factors. One possible reason for this difference is provider familiarity with treating HCV, which likely impacts patient education and counseling, as well as patient knowledge about HCV. Although national efforts have been made to expand HCV treatment to other specialties [30, 31] treating HCV is largely done by hepatologists and primary care providers. Society guidelines influence provider practice as well. International Liver Transplantation Society guidelines from 2017 recommend HCV NAT+ transplantation in HCV negative recipients [32], while, for example, international kidney transplantation guidelines remained hesitant to recommend HCV NAT+ transplantation in HCV negative recipients [27]. One 2020 study documented that only 58% of kidney transplant providers offered HCV NAT+ transplant to HCV negative patients [33]. These factors may lead transplant hepatologists to more routinely recommend HCV NAT+ organs to their patients compared to other specialties. Although consent is document as yes/no in our transplant database, we did not have confirmation that all patients were offered and educated about HCV NAT+ organs. This represents a potential future direction to increase consent rates at our center.

Differences in consent by organ type may also be impacted by different risks and benefits. Our study suggests that patients listed for liver transplant may have the most to benefit from consenting to receive HCV NAT+ organs since patients who do not consent are 33% less likely to be transplanted at any given time compared to patients who do consent. Although we did not find a significant difference in time to transplant for patients listed for a kidney or heart transplant, patients listed for a lung transplant who did not consent to receive HCV NAT+ organs were more likely to receive a transplant compared to patients who did consent. These findings raise the question of whether there is uncaptured confounding factors present, such as disease or symptom severity and baseline HCV disease status contributing to different motivating reasons for patients to consent in different organ fields. Our own data had such a high degree of missingness (72%) for baseline HCV NAT/Ab status that this was difficult to assess. For example, one study exploring lung transplant recipient attitudes toward HCV NAT+ consent documented that patient consent was frequently driven by feelings of desperation and worsening symptom severity [26], suggesting that more severe symptom burden may have led to patient consent in these cases. Two studies interviewing kidney transplant recipients found that patients consented to receive HCV NAT+ organs because of their physician’s recommendation and the perceived shorter time on the waitlist [34, 35]. Limited data are available on patients’ perspectives in other fields. The difference in consent by organ type could also be due to non-standardized program-specific eligibility criteria for HCV NAT+ transplants [33]. Notably, race/ethnicity was not significantly associated with HCV NAT+ consent overall but was associated with time to transplant for some subgroups of patients. Certain findings such as decreased rates of transplant for Non-Hispanic Black patients controlling for HCV NAT+ consent follow well-documented trends in the literature [36]. Future studies should include standardized assessment of patient attitudes and beliefs around HCV NAT+ organs, and whether there are discrepancies between patient and provider assessment of whether HCV NAT+ organs should be considered.

Finally, a patient’s home location was significantly associated with HCV NAT+ consent with rural patients more likely to consent compared to urban patients. Studies have demonstrated geographic disparities in referral, waitlisting, and transplant across organ types with rural patients consistently having less access to care compared to urban patients [37–41]. One possible explanation for this finding is that delays in care for rural patients likely leads to increased disease or symptom severity at time of transplant evaluation [38]. This may then impact the urgency of transplantation, and therefore counseling and patient perceptions surrounding accepting HCV NAT+ organs; however, more data are needed on associations between disease severity and HCV NAT+ consent.

Limitations of this study include the single-center design; as a consequence, results may not be generalizable. Validation of our findings using data from other transplant centers would be an important next step. Second, we were notably unable to distinguish between patients who did not consent to receive HCV NAT+ organs and patients whose medical team recommended against HCV NAT+ organs, introducing a confounding factor in our sample. Similarly, while our focus was on factors associated with HCV NAT+ consent including the relationship between time to transplant, we did not capture other variables that may be important influencers of time to transplant including disease severity, comorbid conditions, organ size, and HCV NAT+ organ eligibility as determined by their transplant team. Future studies should include a more nuanced analysis of how HCV NAT+ relates to time to transplant controlling for factors that influence transplant priority. Fourth, there was a high degree of missingness in patients’ baseline HCV Ab/NAT status at the time of consent, which may influence patients’ decisions on consent to receive an HCV NAT+ organ as our selective sample of data suggests with 60% of HCV Ab+ patients consenting compared to 47% of HCV Ab− patients. Fifth, we may miss intra-patient variation in consent if patients changed their consent over time through multiple listings on the waitlist since this study examined consent only at a patient’s first waitlisting within our study period. Finally, our study did not capture the number of specific providers for each organ type who were responsible for educating and consenting patients within each specific specialty, and therefore particular provider practices may bias our results. Subsequent work should explore provider perceptions and practices on HCV NAT+ consent through a mixed-methods study in conjunction with patient consent data.

In conclusion, our study is one of the first to our knowledge to characterize factors associated with consent for HCV NAT+ organs. We found that English-preferring patients are more likely to consent to receive HCV NAT+ organs, and that organspecific differences exist in patient consent to receive HCV NAT+ organs, with patients listed for liver transplant much more likely to consent compared to other solid organs. However, our study also suggests that the benefits of consenting to receive HCV NAT+ organs may not be consistent across organ types. Overall, as consent rates increase annually, more efforts are needed to ensure that all patients who may benefit from consenting for HCV NAT+ organs are appropriately educated in their language of choice on the possible risks and benefits.

## Figures and Tables

**TABLE 1 |:** Cohort characteristics by HCV NAT+ consent.

	No (*N* = 1559)	Yes (*N* = 1229)	Total (*N* = 2788)	*p* value
**Waitlist age**				
Median	56.0	56.0	56.0	0.997
**Sex**				
Female	578 (56.1%)	452 (43.9%)	1030 (36.9%)	0.872
Male	981 (55.8%)	777 (44.2%)	1758 (63.1%)	
**Race/ethnicity**				
Hispanic/Latino	53 (65.4%)	28 (34.6%)	81 (2.9%)	0.059
NH Black	564 (57.0%)	426 (43.0%)	990 (35.5%)	
NH Other	70 (61.9%)	43 (38.1%)	113 (4.1%)	
NH White	801 (55.0%)	656 (45.0%)	1457 (52.3%)	
Unknown/Declined	71 (48.3%)	76 (51.7%)	147 (5.3%)	
**Preferred language**				
English	1524 (55.5%)	1221 (44.5%)	2745 (98.5%)	0.001
Not English	35 (81.4%)	8 (18.6%)	43 (1.5%)	
**Urban/Rural location** ^a^				
Rural	325 (50.9%)	314 (49.1%)	639 (23.2%)	0.005
Urban	1210 (57.1%)	908 (42.9%)	2118 (76.8%)	
**Marital status**				
Partnered	1008 (56.0%)	791 (44.0%)	1799 (64.5%)	0.768
Single	522 (55.7%)	416 (44.3%)	938 (33.6%)	
Unknown	26 (51.0%)	25 (49.0%)	51 (1.8%)	
**BMI**				
Mean (SD)	28.6 (5.7)	29.4 (5.7)	28.9 (5.7)	<0.001
**Insurance**^b^ Medicaid	42 (51.2%)	40 (48.8%)	82 (3.0%)	0.060
Medicare	901 (56.7%)	688 (43.3%)	1589 (58.1%)	
Other	50 (44.2%)	63 (55.8%)	113 (4.1%)	
Private/Commercial	523 (55.1%)	427 (44.9%)	950 (34.7%)	
**Organ**				
Heart	257 (61.8%)	159 (38.2%)	416 (14.9%)	<0.001
Kidney	830 (58.0%)	602 (42.0%)	1432 (51.4%)	
Liver	156 (32.8%)	319 (67.2%)	475 (17.0%)	
Lung	316 (68.0%)	149 (32.0%)	465 (16.7%)	
**Waitlist year**				
2018	266 (68.2%)	124 (31.8%)	390 (14.0%)	<0.001
2019	351 (54.5%)	293 (45.5%)	644 (23.1%)	
2020	383 (60.0%)	255 (40.0%)	638 (22.9%)	
2021	410 (53.5%)	357 (46.5%)	767 (27.5%)	
2022	149 (42.7%)	200 (57.3%)	349 (12.5%)	
**Highest education** ^c^				
High school or less	358 (54.7%)	296 (45.3%)	654 (29.9%)	0.805
More than high school	832 (54.2%)	704 (45.8%)	1536 (70.1%)	

a Thirty-one patients with missing data were excluded from analysis.

b Fifty-four patients with missing data were excluded from analysis.

c Five hundred and ninety-eight patients with missing data were excluded from analysis.

**TABLE 2 |:** Fine-gray competing risk survival model for HCV NAT+ consent by organ type.

Description	Point estimate	95% Wald confidence limits	
HCV NAT No versus Yes for Heart	1.120	0.861	1.455
HCV NAT No versus Yes for Kidney	0.935	0.803	1.088
HCV NAT No versus Yes for Liver	0.674	0.538	0.844
HCV NAT No versus Yes for Lung	1.637	1.262	2.123

## Abbreviations:

Ab: antibody
HCV: hepatitis C
HCV NAT+: hepatitis C nucleic acid test positive
IQR: interquartile range
NAT: nucleic acid test
UNOS: United Network for Organ Sharing
